# Electric field and aging effects of uniaxial ferroelectrics Sr_*x*_Ba_1−*x*_Nb_2_O_6_ probed by Brillouin scattering

**DOI:** 10.1038/s41598-017-10985-9

**Published:** 2017-09-14

**Authors:** M. Aftabuzzaman, M. A. Helal, R. Paszkowski, J. Dec, W. Kleemann, S. Kojima

**Affiliations:** 1 Graduate School of Pure and Applied Sciences, University of Tsukuba, Tsukuba, Ibaraki 305-8573 Japan; 2 Department of Physics, Pabna University of Science and Technology, Pabna 6600, Bangladesh; 3 Department of Physics, Begum Rokeya University, Rangpur, Rangpur 5400, Bangladesh; 4 Institute of Materials Science, University of Silesia, PL-40-007 Katowice, Poland; 5 Angewandte Physik, Universität Duisburg-Essen, D-47048 Duisburg, Germany

## Abstract

Static and dynamic heterogeneity of disordered system is one of the current topics in materials science. In disordered ferroelectric materials with random fields, dynamic polar nanoregions (PNRs) appear at Burns temperature and freeze into nanodomain state below Curie temperature (*T*_C_). This state is very sensitive to external electric field and aging by which it gradually switches into macrodomain state. However, the role of PNRs in such states below *T*_C_ is still a puzzling issue of materials science. Electric field and aging effects of uniaxial ferroelectric Sr_*x*_Ba_1−*x*_Nb_2_O_6_ (*x* = 0.40, SBN40) single crystals were studied using Brillouin scattering to clarify the critical nature of PNRs in domain states below *T*_C_. On field heating, a broad anomaly in longitudinal acoustic (LA) velocity at low temperature region was due to an incomplete alignment of nanodomains caused by the interaction between PNRs. A sharp anomaly near *T*_C_ was attributed to the complete switching of nanodomain to macrodomain state owing to the lack of interaction among PNRs. After isothermal aging below *T*_C_, the noticeable increase of LA velocity was observed. It was unaffected by cyclic temperature measurements up to *T*_C_, and recovered to initial state outside of a narrow temperature range above and below aging temperature.

## Introduction

The Pb-based relaxor feroelectrics (REFs) with ABO_3_-type perovskite structure such as Pb(Mg_1/3_Nb_2/3_)O_3_ (PMN), (1−*x*)Pb(Mg_1/3_Nb_2/3_)O_3_-*x*PbTiO_3_ (PMN-*x*PT), Pb(Zn_1/3_Nb_2/3_)O_3_ (PZN), (1−*x*)Pb(Zn_1/3_Nb_2/3_)O_3_-*x*PbTiO_3_ (PZN-*x*PT) have attracted much attention owing to their outstanding piezoelectric and electromechanical properties^1–4^. Due to their exceptional piezoelectric effect, these REFs are very useful for various applications in piezoelectric devices. During the last several decades, Pb-based perovskite REFs have been extensively studied, whereas the understanding of Pb-free uniaxial relaxors is still unclear. Recently, the intensive research on Pb-free materials has been triggered due to their emerging demand in green technology.

Uniaxial REFs with tetragonal tungsten bronze (TTB) structure such as Sr_*x*_Ba_1−*x*_Nb_2_O_6_ (SBN) are technologically important materials owing to their remarkably high dielectric, piezoelectric, pyroelectric, and photorefractive properties^5–12^. These excellent physical features are useful for modern applications such as sensors, data storage^8, 11, 13–16^, lasers, and holography^17, 18^. The unique combination of physical properties and Pb-free nature makes SBN single crystals crucial materials for research. It was suggested that smaller lattice parameters of SBN compared to other TTB ferroelectrics is one of the factors responsible for the high values of spontaneous polarization and electro-optic coefficients^19^. SBN undergoes a ferroelectric phase transition from high-temperature nonpolar 4/*mmm* to low-temperature polar 4*mm* tetragonal symmetry^5^. Therefore, the spontaneous polarization of SBN has only one single component along the tetragonal c-axis. The general formula of unit cell of TTB structure can be represented by (A1)_2_(A2)_4_(C)_4_-(B1)_2_(B2)_8_O_30_ with corner sharing distorted BO_6_ octahedra^20^. It is believed that the off-center displacements of B-site ions along the c-axis induce the ferroelectricity in the TTB structure^21–23^. It is also observed that the degree of disorder in ionic occupancy at the A-sites, which is mostly affected by the size and charge of ions, significantly perturb the polar BO_6_ octahedral unit. Hence, the differences of ionic radius and charge at the A-sites ions cause the oxygen octahedral tilting/rotation and induce the characteristic dielectric and ferroelectric behaviors in TTB compounds^22–26^. This octahedral distortion is related to three types of interstitial positions consisting of two square A1-sites, four pentagonal A2-sites, and four trigonal C-sites^27^. In SBN, the A1-sites are occupied only by Sr ions and the A2-sites are occupied by both Ba and Sr ions, while the C-sites and one-sixth of all the A-sites (A1 + A2 -sites) remain unoccupied. These unoccupied A-sites give rise to missing charges with an effective disorder, which are believed to be the most intense sources of quenched random fields (RFs). With increasing Sr/Ba ratio, the disorder at the A-sites increases. This causes an increase of the strength of RFs and an enhancement of the relaxor nature in SBN^8, 28, 29^. The nonequivalent A and B-sites and an extra C-site provide a huge compositional flexibility, which offers extra degrees of freedom for manipulating the TTB structure^30, 31^. The relaxor behavior of SBN has been described on the basis of the random field Ising model^32, 33^. Since the relaxor nature is associated with the charge disorder owing to the unfilled structure^33, 34^, the compositional dependence of the lattice entropy has been explained as a function of Sr/Ba ratio by statistical models of the vacancies at the A-sites^28, 35^. It is observed that in the Sr-rich region the relaxor behavior dominates, especially in the dielectric and elastic properties^35, 36^.

Generally in REFs, the relaxor nature is characterized by the polar nanoregions (PNRs) which play the dominant role in the precursor phenomena of a ferroelectric phase transition. Upon cooling from high temperature, fluctuations of the RFs induce the dynamic PNRs at the Burns temperature *T*_B_. With further cooling from *T*_B_, the PNRs start to grow and a dynamic to static transition of PNRs takes place at the intermediate temperature T* below which the static PNRs grow rapidly. Therefore, it is considered that the PNRs play a vital role in the relaxor behavior by inducing the diffusive and frequency-dispersive dielectric anomalies and various precursor phenomena^37, 38^.

At the Curie temperature *T*_C_, most of the dynamic PNRs are frozen into a nonequilibrium nanodomain state, while the RFs prevent the growth of macrodomains^39^. However, during an aging process of TTB relaxors below *T*_C_, the nonequilibrium nanodomain state gradually changes into a metastable macrodomain state with opposite spontaneous polarization direction^40, 41^. After several years aging below *T*_C_, a stable macrodomain or even a single domain state can be obtained^39^. Aging is a universal phenomenon in disordered systems such as spin glasses, super-cooled liquids, polymers, and REFs^42^. Aging of the susceptibility components of highly disordered SBN75 with strong RFs shows rejuvenation and memory effects, which indicate the possible glassy nature of the low-temperature ground state^43, 44^. The compositional inhomogeneity in the nanoscale regions of these crystals induces the RFs, which cause a diffused phase transition and the formation of nanodomain structure below *T*_C_, while the growth of the macro/single domain state is restricted by RFs^39^. However, by applying an external electric field, the single domain state has been observed in polar cut SBN61 single crystal^39, 45^.

Most of the experimental efforts have been made to study the uniaxial TTB REFs mainly focusing on their different functional properties, structures, and origin of the relaxor nature and its dependence on the composition. The existence of the characteristic temperatures, *T*_B_ and T*, have been confirmed in SBN75 by acoustic emission, and the electric field effect on the phase transition has been reported^46^. By the low temperature acoustic emission, a field induced orthorhombic phase is observed within the modulated incommensurate tetragonal structure of SBN75^47^. The crystal structures of SBN and other REFs with TTB structure such as Ca_*x*_Ba_1−*x*_Nb_2_O_6_ (CBN) have been reinvestigated using an automated electron diffraction tomography method with beam precession to confirm the tetragonal symmetry with space group *P*4*bm* at room temperature^48^. The appearance of quasi-static precursor polar regions near *T*_C_ in the paraelectric phase has been observed by piezoresponse force microscopy (PFM)^41^ and Raman scattering^49^. The relaxor nature of SBN61 has been studied by Brillouin scattering, dielectric, and pyroelectric measurements and from the nonlinear dielectric responses, the increase of the size of PNRs into long range domains is observed near and below *T*_C_^50–52^. The field induced single domain state and spontaneous back switching are observed in SBN61^39, 45^. Recently, a CBN30 single crystal has been studied by Brillouin scattering under dc electric field^53^. In the ferroelectric phase, an incomplete alignment of nanodomains and enhancement of the long-range ferroelectric order are observed. In addition, the persistence of a coexisting state of nanodomains and macrodomains is observed up to a high electric field due to the incomplete switching of nanodomains. However, the reason of this incomplete alignment/switching is unclear.

The nanodomain state of ferroelectric materials is very sensitive to an external electric field and aging by which it gradually switches into a metastable macrodomain state. As a result, the elastic properties of materials such as sound velocity and sound attenuation are changed. The Brillouin scattering spectroscopy is a powerful tool to observe the frequency and width of acoustic phonon modes which are proportional to the sound velocity and sound attenuation respectively. Therefore, the use of Brillouin scattering is a new approach of experimental technique to study the aging and electric field effect of the ferroelectric materials. Since RFs always try to stabilize the nanodomain state by restricting the formation of macrodomains^39^, the stability of nanodomain state depends on the strength of RFs. SBN40 single crystal is a suitable material to study the switching of nanodomain state below *T*_C_ due to uniaxial nature of the spontaneous polarization and the presence of weak RFs that can be suppressed by a small amount of external electric field.

Therefore, in the present study, the elastic properties of SBN40 single crystals have been investigated under zero and externally applied dc electric field using the broadband Brillouin scattering spectroscopy to clarify the critical nature and related functionality of PNRs and domain states below *T*_C_. In addition, experiments on aging and its temperature dependence have also been performed which will give more insights into the understanding of microscopic nature of domain state in a ferroelectric phase.

## Results and Discussion

### Effects of temperature

**Figure 1:**
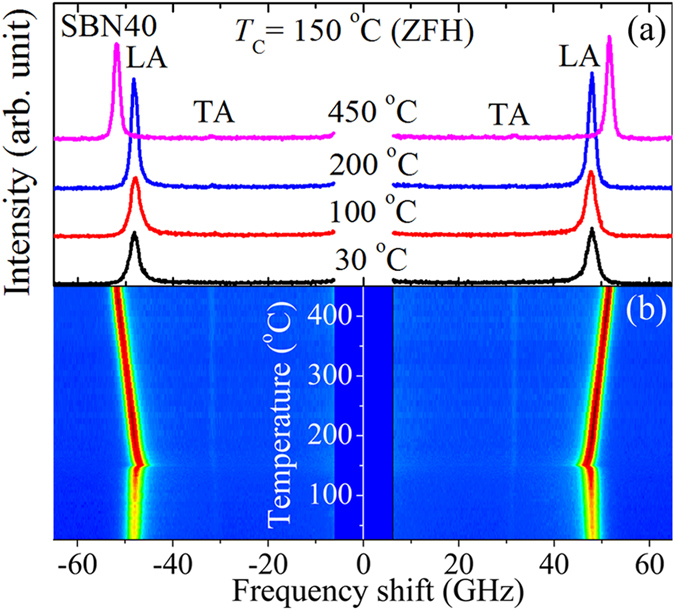
(**a**) Brillouin scattering spectra of SBN40 (*c*-plate) at some selected temperatures under ZFH. (**b**) Contour map of the scattering intensity vs. temperature and frequency shift (red and blue color indicate the high and low intensities respectively). The elastic scattering was removed near 0 GHz.

**Figure 2:**
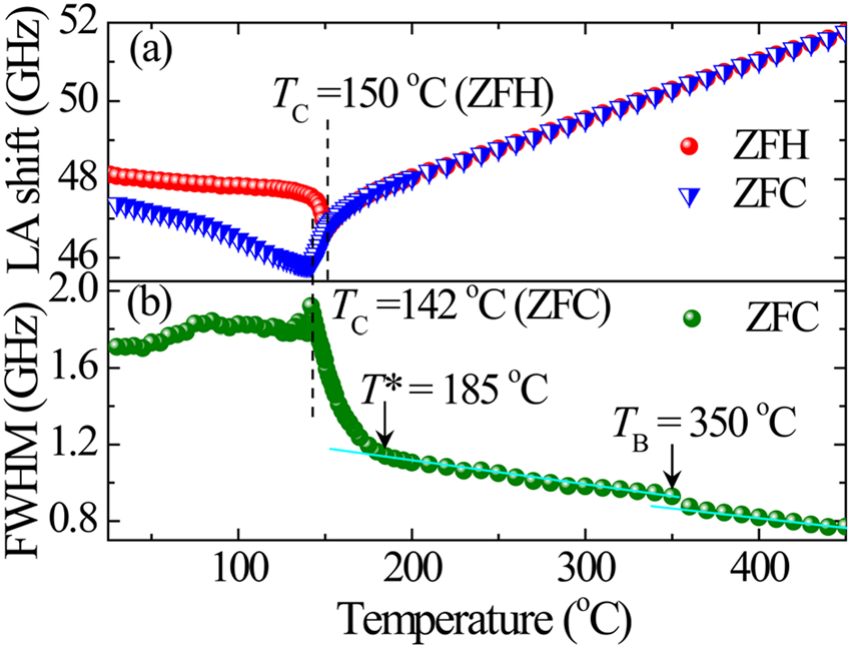
Temperature dependences of (**a**) the Brillouin shift on ZFH and ZFC and (**b**) FWHM on ZFC.

Figure 1(a) shows the observed typical Brillouin scattering spectra of the SBN40 (*c*-plate) single crystal at some selected temperatures measured at *c*(*a*, *a* + *b*)$\mathop{c}\limits^{\bar{} }$ scattering geometry upon a zero field heating (ZFH) process. The contour map of the Brillouin scattering intensity vs. temperature and frequency shift is shown in Fig. 1(b), where the elastic scattering was removed in the vicinity of 0 GHz. These observed spectra consist of the Brillouin peak doublets attributed to the scattered longitudinal acoustic (LA) and transverse acoustic (TA) phonon modes near the Brillouin zone center. From Fig. 1, it is clearly seen that a very weak TA peak persists in the entire temperature range from 26 to 450 °C. According to the selection rules, the TA mode is not allowed for tetragonal 4*mm* or 4/*mmm* point group in back scattering geometries except in *c*(*ab*)$\bar{c}$ ^50^, which is equivalent to the *c*(*a*, *a* + *b*)$\mathop{c}\limits^{\bar{} }$ geometry. Therefore, the existence of the TA mode in all temperature regions above and below *T*_C_ observed at *c*(*a*, *a* + *b*)$\mathop{c}\limits^{\bar{} }$ geometry indicates that the SBN40 single crystal belongs to the tetragonal symmetry both in paraelectric and ferroelectric phases. The measured Brillouin spectra were fitted using the Voigt functions, a convolution of Lorentzian and Gaussian functions at which the width of Gaussian function was fixed as an instrumental function, to obtain the Brillouin shift *ν*_B_, the full width at half maximum (FWHM) *Γ*_B_, and the peak intensity of the phonon modes. The *ν*_B_ and FWHM of the LA mode were plotted as functions of temperature as shown in Fig. 2. A much sharper minimum of *ν*_B_ was observed at 150 °C on ZFH and at ~142 °C on zero field cooling (ZFC) as shown in Fig. 2(a). Below *T*_C_, the noticeable difference between ZFH and ZFC is due to the metastable irreversible domain structure caused by an incomplete switching of nanodomains induced by quenched RFs during the cooling process^54, 55^. The relatively sharp phase transition on ZFH and a remarkable thermal hysteresis observed in the LA shift between ZFH and ZFC processes indicate the presence of relatively weak RFs in the SBN40 single crystal. Similar acoustic hysteresis behavior was reported in other REFs^39, 55–57^.

**Figure 3:**
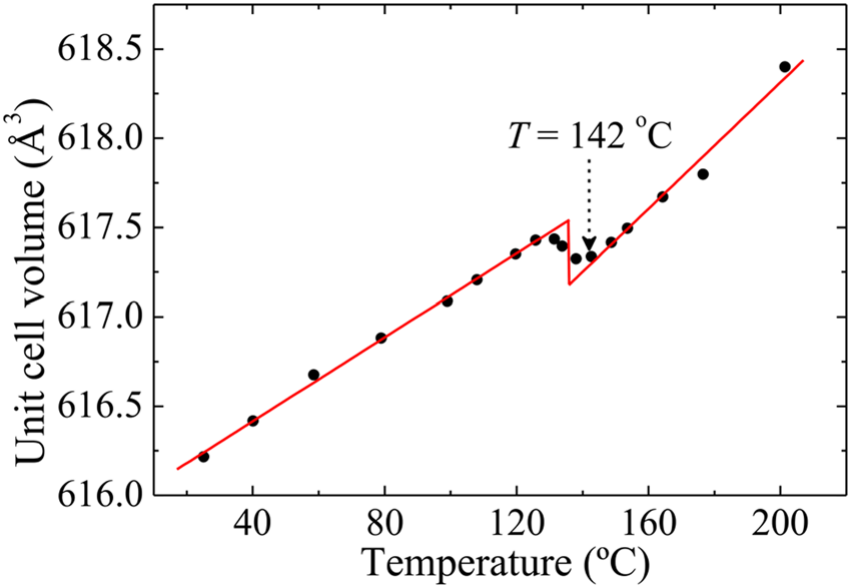
Temperature dependence of unit cell volume of SBN40 single crystal on ZFH.

The elastic properties are sensitive to the characteristic temperatures of PNRs and show anomalies at these temperatures via the scattering of LA phonons by PNRs^58^. Therefore, the FWHM of LA mode, which is directly related to the sound attenuation, was measured in the wide temperature range between 26 and 450 °C as shown in Fig. 2(b). Upon cooling from a high temperature, the FWHM shows a deviation from its linear change near 350 °C due to the appearance of dynamic PNRs which scatter the LA phonons and cause an increase of the LA width. A deviation from the linear temperature dependence was also observed at 185 °C and below this temperature, a rapid increase in the LA width was observed due to the dynamic-to-static transition of PNRs and scattering of the LA phonons by static PNRs^59^. Therefore, these anomalies can be attributed to the characteristic temperatures of PNRs, i.e., *T*_B_ = 350 °C and T* = 185 °C. It is observed that the values of *T*_B_ and T* are very close to those of SBN61 and SBN75^39, 46^, and seem to be common for all compositions of SBN. Similar behavior was also observed in Pb-based REFs with perovskite structure^60^. The increase of the scattering of LA mode by PNRs is stopped at *T*_C_, because most of the PNRs become frozen into the ferroelectric nanodomain structure. *T*_C_ = 142 °C and 150 °C were determined from the temperature dependence of FWHM of the LA mode on ZFC and ZFH processes, respectively. The temperature dependence of the unit cell volume (Fig. 3) showed a clear deviation at *T* = 142 °C from linearity of high temperature region. It indicates the structural transition of SBN40 single crystal from high-temperature nonpolar 4/*mmm* to low-temperature polar 4*mm* tetragonal symmetry. This result is in good agreement with the Brillouin scattering measurement, and this temperature is identified as *T*_C_ = 142 °C on ZFC as shown in Fig. 2(b), while *T*_C_ = 150 °C is observed on ZFH. The small discrepancy in temperature may be due to the different procedures of experiments.

**Figure 4:**
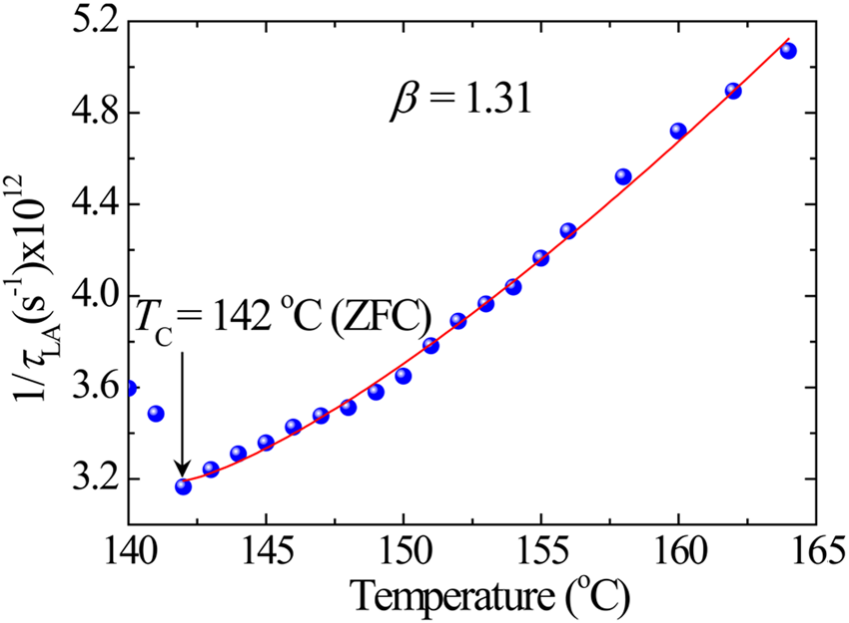
Temperature dependence of the inverse relaxation time shows the stretched slowing down.

The relaxation time of LA mode, *τ*_LA_ was determined using^61, 62^

1 $${\tau }_{{\rm{LA}}}=\frac{{{\rm{\Gamma }}}_{{\rm{{\rm B}}}}-{{\rm{\Gamma }}}_{\infty }}{2\pi ({{\nu }^{2}}_{\infty }-{{\nu }^{2}}_{B})},$$

where *Γ*_∞_ is the background damping obtained in terms of FWHM at the highest temperature in Fig. 2(b) i.e., in the present case the value of *Γ*_∞_ is 0.77 GHz at 450 °C, and *ν*_∞_ is the LA shift at a very high temperature region (>*T*_B_), where its temperature dependence is linear^62^. In a very high temperature region above *T*_B_, ferroelectric materials are in a paraelectric phase without any PNRs. In this region, a linear temperature dependence of* ν*_B_ is observed only due to lattice anharmonicity^63^. In order to obtain *ν*_∞_, the high temperature linear part of *ν*_B_ above *T*_B_ was fitted by a linear function *ν*_∞_ (*T*) = 45.36 + 0.01421 × *T* (GHz). It should be noted that the *τ*_LA_ is not sensitive to the choice of *ν*_∞_. Even if the highest value of *ν*_B_ at 450 °C is used as *ν*_∞_, almost the same *τ*_LA_ is obtained. The temperature dependence of the inverse of *τ*_LA_ was shown in Fig. 4, where the solid line shows the best fitted curve using^64^

2 $$\frac{1}{{\tau }_{{\rm{LA}}}}=\frac{1}{\,{\tau }_{0}}+\frac{1}{\,{\tau }_{1}}{(\frac{T-{T}_{C}}{{T}_{C}})}^{\beta },(1\le \beta )\,{\rm{for}}\,T > {T}_{C},$$

where the stretching index *β* = 1.0 corresponds to the normal critical slowing down without RFs, while *β* > 1.0 corresponds to the stretched slowing down of the relaxation time due to the increase of the strength of RFs^65^. Best fitting of inverse *τ*_LA_ using Eq. (2) (Fig. 4) yields *τ*_0_ = 0.31 ps and *τ*_1_ = 0.04 ps, while *β* = 1.31 indicates that SBN40 single crystal exhibits the stretched critical slowing down of PNRs. Since *β* = 3.0 was reported for PZN-7PT^64^, SBN40 single crystals are ferroelectrics with comparatively weak RFs. As a result, a very weak frequency dependence of dielectric susceptibility was observed and suggested that the phase transition is weakly first order and SBN40 is a crossover material from normal to REFs^66^.

### Effects of electric field

**Figure 5:**
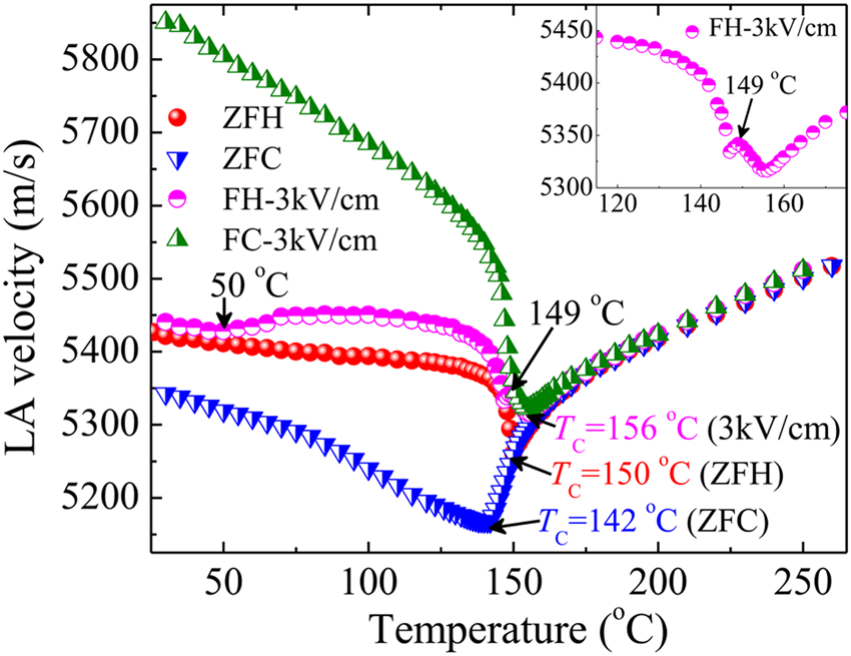
Temperature dependence of LA velocity under 3 kV/cm electric field along the [001] direction.

The LA velocity (*V*_LA_) was determined from the *ν*_B_ using the equation *V*_LA_ = λ*ν*_B_/2*n*, where *λ* is the wavelength of the incident laser light (532 nm at the present case) and *n* is the ordinary refractive index of the sample at *λ*. The value of the *n* for a SBN40 single crystal at the incident laser wavelength of 532 nm is 2.363^67^. Figure 5 shows the temperature dependence of *V*_LA_ under zero and 3.0 kV/cm electric field along the [001] direction on heating and cooling processes. On field heating (FH) from *T* = 25 °C under 3.0 kV/cm, a broad and weak anomaly in *V*_LA_ around 50 °C was observed. It suggests an incomplete alignment of nanodomains due to the interaction between PNRs^43, 68^. Upon further heating, a sharp and small increase in *V*_LA_ around 149 °C (inset of Fig. 5) was observed due to a complete switching of the nanodomain state induced by the RFs into the macrodomain/single domain state induced by the external electric field. A similar acoustic anomaly was observed in a CBN30 single crystal at a low temperature region^53^, while the anomaly near *T*_C_ was not observed. Presumably the applied field was not sufficient for overcoming the RFs and switching the nanodomains. In a low temperature region, PNRs are strongly correlated with each other, hence the alignment of nanodomains under the external electric field becomes restricted. When the temperature increases towards *T*_C_, the correlation among PNRs becomes sluggish, therefore, under a sufficiently high electric field, the alignment of nanodomains/static PNRs becomes facilitated and enables complete switching into the macro/single domain state. On subsequent continuous field cooling (FC) under 3.0 kV/cm, the anomaly at 149 °C was absent and a remarkable increase of the *V*_LA_ was observed in the ferroelectric phase because of the complete suppression of nanodomains during the previous FH process. Therefore, the FC curve is actually attributed to the field induced macro/single domain state.

### Effects of aging

Upon cooling, the disordered ferroelectrics containing random charges and random ionic radii such as PMN freeze out into a glassy state^69, 70^, while SBN containing the random cation vacancies, transforms into a metastable domain state induced by RFs below *T*_C_. The metastability of domains and pinning of the domain wall configurations by RFs cause a very slow ‘aging’ dynamics of the structure, which drives it towards the equilibrium^44^. When disordered materials approach thermal equilibrium, this subsides into states of progressively lower free energy, i.e., states of a lower electrical or magnetic susceptibility^71^. Although aging is a common feature of disordered materials^42^, which causes a variety of metastable states, the nature of aging is different for the different types of disordered systems under variant temperature and field histories^72^. Dec *et al*.^44^ reported that the dielectric susceptibility of uniaxial relaxor SBN75 single crystals shows a hole-like aging in the ferroelectric domain state. After isothermal aging, both rejuvenation and memory effects were also observed in temperature cycle experiments up to *T*_C_, which reflects the presence of cluster glass-like disorder of a complex domain structure because of the strong RFs. Recently, Kleemann *et al*.^43, 70, 73^ observed similar effects not only in heterovalent (SBN75, PMN) systems with strong RFs, but also in isovalent BaTi_1−*x*_Zr_*x*_O_3_ (*x* = 0.35, BTZ35) with weak RFs. They also proposed that these effects might be a universal signature of all REFs^73^.

**Figure 6:**
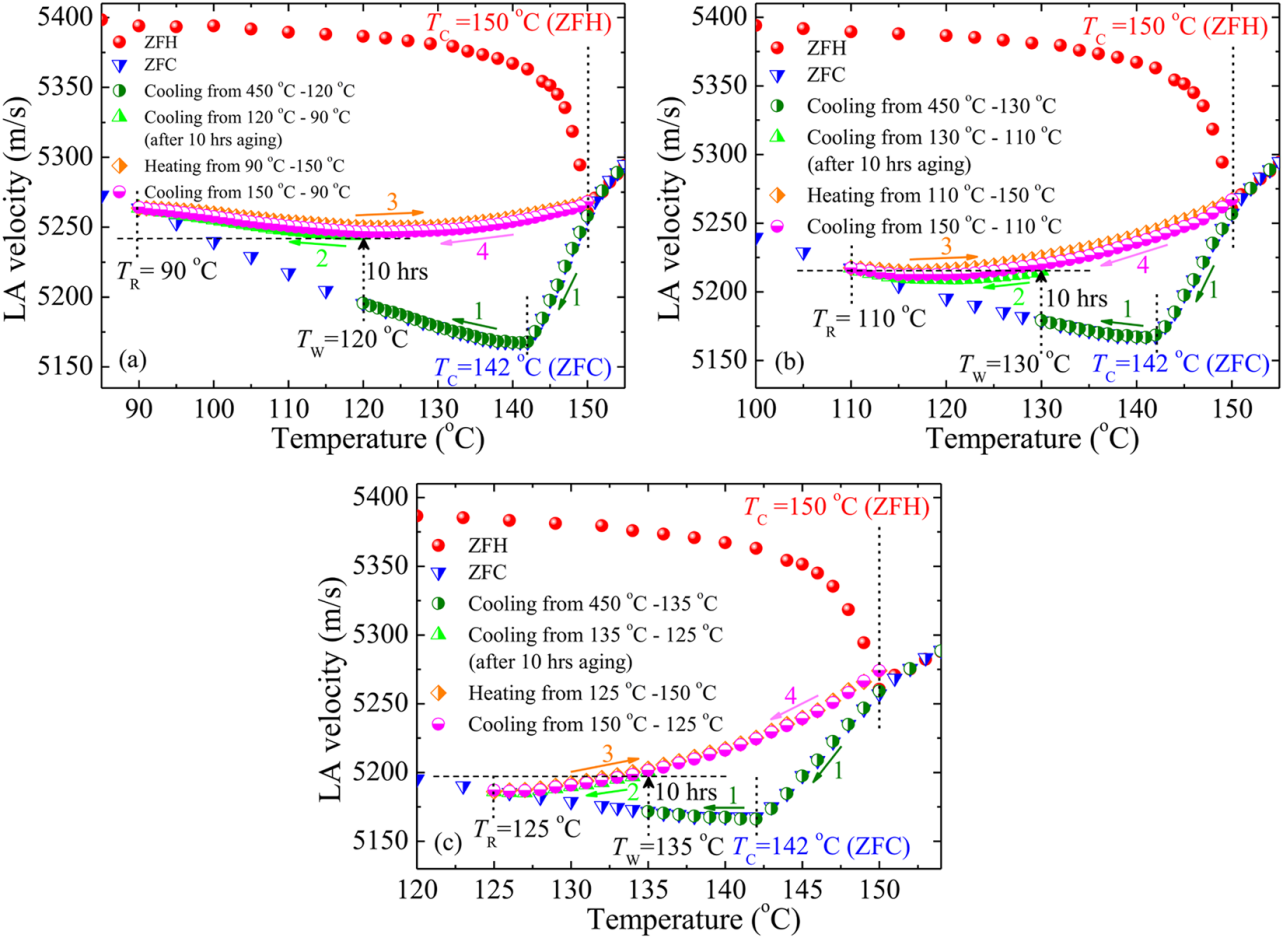
LA velocity of SBN40 vs. temperature after ZFC from 450 °C on first cooling to *T*_W_ (curve 1), then aging for 10 h at (**a**) *T*_W_ = 120 °C, (**b**) *T*_W_ = 130 °C, and (**c**) *T*_W_ = 135 °C and cooling down (curve 2) until it merges with a ZFC reference curve at *T*_R_, continuous reheating up to 150 °C (curve 3), and subsequent continuous cooling back to *T*_R_ (curve 4). Red circle and blue triangle are reference curves measured on ZFH and ZFC, respectively without aging.

In the present study, an isothermal aging experiment on SBN40 single crystals with weak RFs was performed in the “ferroelectric” phase. Since during isothermal aging the deep ‘hole’ being burnt at a waiting temperature *T*_W_, indicates the approach of metastable domain states to the equilibrium via lowering dielectric susceptibilities^44^, it is expected that aging at *T*_W_ will also affect *V*_LA_. Figure 6 shows *V*_LA_ of SBN40 vs. temperature measured under the following procedures: (1) first the sample was cooled from 450 °C to *T*_W_ on ZFC (curve 1), (2) then aging at (a) *T*_W_ = 120 °C, (b) *T*_W_ = 130 °C, and (c) *T*_W_ = 135 °C for 10 h and cooling down (curve 2) to a certain temperature *T*_R_, where *V*_LA_ merges with the ZFC reference curve, (3) continuous reheating up to 150 °C (curve 3), and (4) subsequent continuous cooling back to *T*_R_ (curve 4). Red circles and blue triangles are reference curves measured on ZFH and ZFC, respectively without aging. During isothermal aging below *T*_C_, the growth into a macrodomain state occurs from the nonequilibrium nanodomain state^40, 41^, while RFs try to stabilize the nanodomain state and suppress the formation of the macrodomains^39, 53^. Therefore, after aging the sample at *T*_W_ for 10 h under zero field, an increase of *V*_LA_ was observed. From Fig. 6(a), one can assume that it is an irreversible growth into ordered domain or cumulative aging as observed in classical ferroelectrics. But in Fig. 6(b) and (c), it was clearly observed that on cooling from *T*_W_, the *V*_LA_ start to decrease (curve 2) and recover to the unaged state at *T*_R_, which excludes the possibility of being cumulative aging. On the other hand, the aged state was unaffected by cyclic temperature measurements up to *T*_C_ and merged with the unaged ZFC curve outside of a narrow temperature range (‘aging window’) above and below *T*_W_. This stable memory effect of aging, which recovers above and below *T*_W_, indicates the existence of the interaction between PNRs. In another aging experiment (see Supplementary Fig. S1), the cooling curve 2 in Fig. 6(b) had been extended to 10 °C below *T*_R_ =110 °C. It was observed that below *T*_R_, all (aged) curves almost completely merge with and follow the ‘unaged’ ZFC reference curve which confirms the complete recovery of the memory. In addition, by extending the heating curve 3 in Fig. 6(b) up to 10 °C above *T*_C_ (=150 °C on ZFH) and subsequent continuous cooling back to 100 °C, about 75% erase of an aging memory at *T*_W_ = 130 °C was observed (see Supplementary Fig. S2). As a result, the sound velocity (cooling curve 4) try to follow the unaged cooling curve 1 and merge with the ‘unaged’ ZFC reference curve at about 5 °C prior to *T*_R_. Upon heating the sample above *T*_C_, a transition from static/frozen to dynamic state of PNRs begins and consequently, the interaction among PNRs becomes weak. Above T*, this transition become completed and all PNRs become dynamic and non-interacting. Therefore, it was suggested that the memory of aging can be erased completely by heating the sample above T*.

In Fig. 6, it was clearly observed that the aging window between *T*_C_ and *T*_R_ significantly decreases to 60, 40, and 25 °C for the increase of *T*_W_ as 120, 130, and 135 °C, respectively. Consequently, by increasing *T*_W_, a remarkable suppression of *V*_LA_ was also observed. Near *T*_C_, the influence of RFs increases, which stabilize the nanodomains by pinning of domain walls and suppress the formation of the macrodomains. In addition, the fluctuations of local polarization of PNRs become stronger in the vicinity of *T*_C_^53^ and, hence, the *V*_LA_ decreases. On the other hand, when *T*_W_ approaches *T*_C_, the interaction among PNRs becomes weaker and the strong RFs make the nanodomains more stable by restricting the formation of macrodomains. As a result, near *T*_C_, the return of the metastable state of *V*_LA_ (at *T*_W_ after 10 h aging) to its initial/unaged nanodomain state (ZFC reference curve) becomes easier by a small temperature variation through *T*_W_. Hence, at a lower *T*_W_, the correlation among PNRs is strong to cause a strong memory, which recovers slowly and results in a larger *T*_C_ − *T*_R_ value. However, at higher *T*_W_ this correlation becomes weaker and causes a weak memory effect, which recovers quickly and results in a smaller *T*_C_ − *T*_R_.

## Conclusions

The effect of an electric field and aging on the LA velocity of a SBN40 single crystal were studied using the broadband Brillouin scattering spectroscopy. Three characteristic temperatures, namely, the Burns temperature *T*_B_ = 350 °C, the intermediate temperature T* = 185 °C, and the Curie temperature *T*_C_ = 150 °C on ZFH and 142 °C on ZFC, were determined from the temperature dependence of the LA width. The temperature dependence of the inverse relaxation time indicates that a SBN40 single crystal with relatively weak RFs exhibits a stretched critical slowing down of PNRs. The effect of the external electric field along the [001] direction was clearly observed. On FH under 3.0 kV/cm, an incomplete alignment of nanodomains at 50 °C due to the interaction between PNRs and a complete switching of a nanodomain to a macrodomain state at 149 °C were observed. On FC, the anomaly at 149 °C was not observed because of the complete switching of nanodomains during the previous FH process. A marked thermal hysteresis was observed below *T*_C_ in the LA shift between ZFH and ZFC processes. It is also related to the incomplete switching of nanodomains induced by quenched RFs. After aging at *T*_W_ under zero field in a ferroelectric phase, an increase of LA velocity was observed, which was unaffected by the cyclic temperature measurements up to *T*_C_ and recovered to the initial state outside of a narrow temperature range above and below *T*_W_. By increasing *T*_W_, a remarkable decrease of aging window was observed due to the weaker interaction between PNRs.

## Methods

The Sr_*x*_Ba_1−*x*_Nb_2_O_6_ (*x* = 0.40, SBN40) single crystals were grown by the Czochralski method^74^. A (001)-oriented plate (*c*-plate) with 5 × 5 mm^2^ surfaces, which were polished to optical quality, and 1 mm thickness was used for measurements. Silver plate electrodes were coated on the surfaces of the crystal with a hole of 1 mm radius on one of the surfaces for the application of a dc electric field along the [001] direction. Brillouin spectra were measured in back scattering geometry using a high-resolution 3 + 3 passes Sandercock-type tandem Fabry–Perot interferometer (JRS TFP-1) combined with an optical microscope (Olympus BX-60) and a single frequency green yttrium aluminium garnet (YAG) laser (Coherent Compass 315M-100) with a wavelength of 532 nm and 100 mW output^65^. The mirror space was set at 2 mm with a free spectral range of 75 GHz. Temperature of the sample was controlled by a heating/cooling stage (Linkam THMS600) with a stability of ±0.1 °C. Before the start of every measurement, the electrodes of a sample were short-circuited for 10 min at a high enough temperature to remove any memory effect of electric field retained from previous treatments.

Temperature dependences of lattice parameters of SBN40 were measured using an X-ray Bond method^75^ with an uncertainty as low as of the order of Δ*d*/*d* = 10^−5^. In order to carry out such precise measurement X-ray metric value of *λ* CuKα_1_^76^ and high quality of single crystals are required. Values of the lattice parameters were extracted from absolute shift of the reflex position (chosen at sufficiently high 2*θ* angle), which was additionally corrected due to systematic uncertainties of the shifts. The calculations of lattice parameters were based on 16,0,0 reflection (*θ* = 80.879°) for an orientation parallel to the tetragonal *c*- axis and 0,0,5 reflection (*θ* = 78.146°) for an orientation perpendicular to the tetragonal *c*- axis. More details related to the measurement procedure was presented elsewhere^77^. All measurements of the lattice parameters were conducted in the air in the temperature range of 20–200 °C. Throughout the measurements the temperature was detected with the help of Ni-CrNi thermocouple and the stability of temperature was better than 0.1 °C. Basing on the temperature dependences of the lattice parameters thus obtained a volume of the SBN40 elementary unit cell as a function of temperature was calculated.

## Electronic supplementary material

Supplementary Figures
