# Supplementary material for: Electric field and aging effects of uniaxial ferroelectrics SrxBa1−xNb2O6 probed by Brillouin scattering
Source: Sci Rep. 2017 Sep 14;7:11615. doi: 10.1038/s41598-017-10985-9 (PMC5599614; doi:10.1038/s41598-017-10985-9)
Supplement: Supplementary file 1 — Supplementary Figures [file 41598_2017_10985_MOESM1_ESM.pdf]

# Electric field and aging effects of uniaxial ferroelectrics $Sr_xBa_{1-x}Nb_2O_6$ probed by Brillouin scattering

M. Aftabuzzaman[1, 2,*], M. A. Helal[3], R. Paszkowski[4], J. Dec[4], W. Kleemann[5] & S. Kojima[1]

[1]Graduate School of Pure and Applied Sciences, University of Tsukuba, Tsukuba, Ibaraki 305-8573, Japan.

[2]Department of Physics, Pabna University of Science and Technology, Pabna, 6600, Bangladesh.

[3]Department of Physics, Begum Rokeya University, Rangpur, Rangpur, 5400, Bangladesh.

[4]Institute of Materials Science, University of Silesia, PL-40-007 Katowice, Poland.

[5]Angewandte Physik, Universität Duisburg-Essen, D-47048 Duisburg, Germany.

[*]azamanphy@gmail.com

## Supplementary Figures

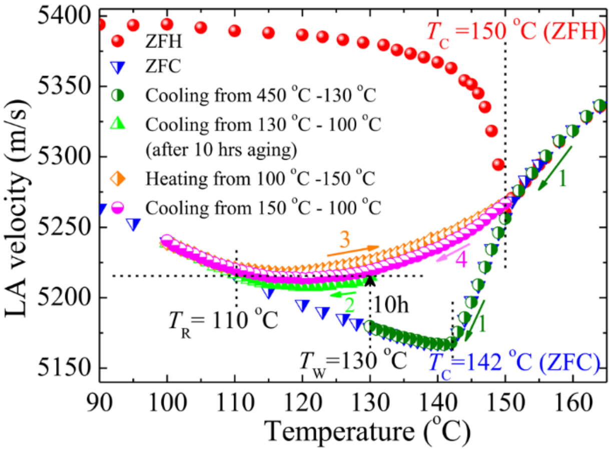


**Supplementary Figure S1.** LA velocity of SBN40 vs. temperature after ZFC from 450 °C on first cooling to $T_W$ = 130 °C (curve 1), then aging for 10 h at $T_W$ and cooling down to 100 °C (curve 2), continuous reheating up to 150 °C (curve 3), and subsequent continuous cooling back to 100 °C (curve 4). Red circle and blue triangle are reference curves measured on ZFH and ZFC, respectively without aging.

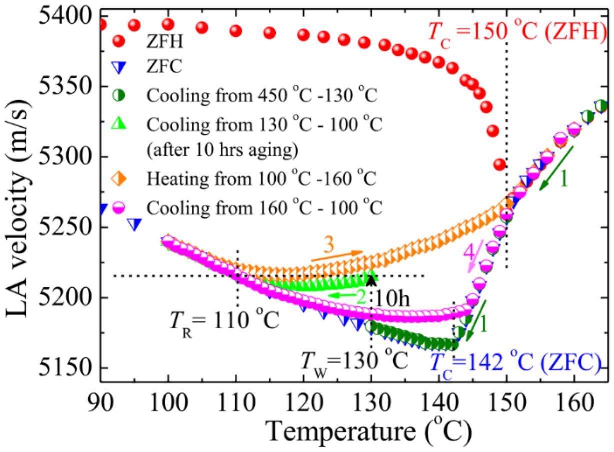


**Supplementary Figure S2.** LA velocity of SBN40 vs. temperature after ZFC from 450 °C on first cooling to $T_W$ = 130 °C (curve 1), then aging for 10 h at $T_W$ and cooling down to 100 °C (curve 2), continuous reheating up to 160 °C (curve 3), and subsequent continuous cooling back to 100 °C (curve 4). Red circle and blue triangle are reference curves measured on ZFH and ZFC, respectively without aging.
